# Posttranslational Chemical Mutagenesis Methods to Insert Posttranslational Modifications into Recombinant Proteins

**DOI:** 10.3390/molecules27144389

**Published:** 2022-07-08

**Authors:** Omer Harel, Muhammad Jbara

**Affiliations:** School of Chemistry, Raymond and Beverly Sackler Faculty of Exact Sciences, Tel Aviv University, Tel Aviv 69978, Israel; tau.omer.harel@gmail.com

**Keywords:** posttranslational modifications, proteins, alkylation, dehydroalanine, cysteine, bioconjugation, late-stage functionalization

## Abstract

Posttranslational modifications (PTMs) dramatically expand the functional diversity of the proteome. The precise addition and removal of PTMs appears to modulate protein structure and function and control key regulatory processes in living systems. Deciphering how particular PTMs affect protein activity is a current frontier in biology and medicine. The large number of PTMs which can appear in several distinct positions, states, and combinations makes preparing such complex analogs using conventional biological and chemical tools challenging. Strategies to access homogeneous and precisely modified proteins with desired PTMs at selected sites and in feasible quantities are critical to interpreting their molecular code. Here, we summarize recent advances in posttranslational chemical mutagenesis and late-stage functionalization chemistry to transfer novel PTM mimicry into recombinant proteins with emphasis on novel transformations.

## 1. Introduction

Posttranslational modifications (PTMs) are dynamic transformations that regulate protein structure and function and impact central biological processes [1]. These dynamic modifications can alter protein stability, interaction, cellular localization, and overall activity. Therefore, they play a central regulatory task in proteome diversity and cell biology. A wide range of PTMs have been reported in the past decades through the use of chemical biology tools coupled with mass spectrometry and bioinformatic techniques [2]. These modifications have been detected in the amino-acid side-chains, polypeptide backbone, and terminus. While the structural role of these modifications can be interpreted and predicted, the functional consequence of many PTMs is still elusive, as the availability of site-specifically modified proteins remains limited [3,4].

**Figure 1:**
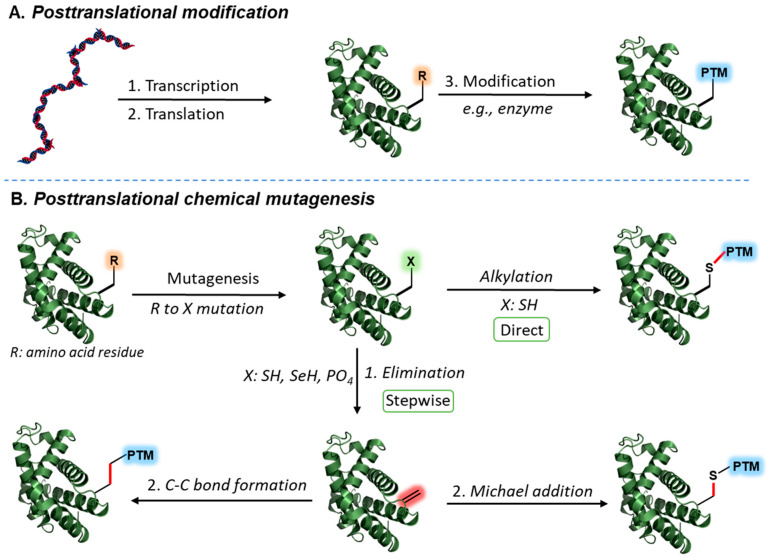
Enzymatic and synthetic posttranslational modification of proteins. (**A**) Posttranslational modification in nature via enzymes. (**B**) Chemoselective installation of posttranslational modification mimics via *S*-alkylation of Cys or dehydroalanine (Dha) functionalization via Michael addition or carbon–carbon bond formation.

Abnormal posttranslational modification of proteins or misregulation of these marks is implicated in many human diseases. Access to homogeneously modified proteins with desired PTMs is critical to interpreting their molecular role and physiological outcome. Unfortunately, investigating a specific PTM poses many challenges. Many PTMs have been discovered at several positions, which could also appear in different combinations to “crosstalk” and yield a new complex phenotypic outcome. In nature, many PTMs are attached enzymatically to proteins in a very controlled manner (Figure 1A). However, in vitro enzymatic modification of isolated proteins is challenging, as it often results in a heterogeneous product due to insufficient specificity and a lack of control over the degree of modification. Other biological approaches, such as genetic code expansion technology, allow site-specific incorporation of unnatural amino acids into any protein of interest in cells using engineered orthogonal aminoacyl-tRNA synthetase/tRNA [5,6]. Importantly, this strategy has enabled selective insertion of several PTMs into recombinant proteins, including methylation, acetylation, nitration, and phosphorylation. Despite the enormous potential of this approach, this method relies on the availability of an orthogonal tRNA synthetase for desired modification, and it is difficult to apply for transferring multiple PTMs into the same protein [5,6].

Chemical protein synthesis provides a powerful means to prepare native and modified proteins in an effective and controlled manner [7,8]. Protein synthesis allows for protein production with selective insertion of virtually any desired PTM to produce modified proteins in high homogeneity [9,10]. In this method, short synthetic peptides are prepared using solid-phase peptide synthesis (SPPS), which allows for the incorporation of the desired PTM during the peptide elongation at the solid support. Next, the produced synthetic peptides are reacted in a stepwise manner in solution using chemoselective ligation approaches to furnish full-length, modified proteins [11,12]. Many effective chemical ligation reactions have been developed in the past decades. The most frequently employed ligation methods are native chemical ligation (NCL) [13], α-ketoacid–hydroxylamine (KAHA) ligation [14], diselenide–selenoester ligation (DSL) [15], and Ser/Thr ligation (STL) [16]. The NCL approach is the most utilized ligation method, owing to the accessibility of the building blocks and the mild reaction conditions. Recent advances in protecting group chemistry [17,18], functional tags [19,20], continuous flow synthesis [21,22], and post-synthetic modifications [7,9] allow for the preparation of large and complex protein targets with a broad range of PTMs for various applications. Moreover, the development of the protein semi-synthesis technique has expanded the scope of modified protein production by combining synthetic peptides and large recombinant protein fragments in solution using intein technology [23,24]. Chemical protein synthesis and semi-synthetic strategies have enabled the production of phosphorylated [25,26], methylated [27,28], glycosylated [29,30], acetylated [31,32], sulfonated [33,34], sumoylated [35,36], and ubiquitinated [37,38] proteins for various applications. Despite the significant advantages of current chemical tools, current methods are usually used to prepare medium-size proteins (~200 AA) or modified proteins at the C- or N-termini [39].

Late-stage protein modification allows for rapid and direct installation of desired transformations into recombinant folded proteins (Figure 1B) [40,41] The low abundancy and unique reactivity of Cys residue within the human proteome rendered it the site of choice for numerous transformations. The high nucleophilicity of the sulfhydryl residue (pKa ∼8) in physiological conditions enables orthogonal transformations in the presence of other reactive residues [42,43], including Cys elimination [44,45], alkylation [46,47], and arylation [48,49]. For example, Cys alkylation is a wildly used approach for chemoselective installation of PTM mimics into proteins pre-engineered with Cys residue at the modification site via site-specific mutagenesis [50]. Another powerful strategy is using the nonproteinogenic residue dehydroalanine (Dha), which has been proven as an effective and reliable approach to chemoselectivity, to incorporate various PTMs into recombinant proteins [51]. In this stepwise method, the protein of interest is usually engineered with Cys/Sec mutation at the desired modification site, followed by residue elimination to provide Dha-tagged proteins at a predetermined site. Finally, the incorporated Dha undergoes a chemoselective Michael-type addition or novel carbon–carbon bond formation reaction to install the desired PTM [51]. Current chemical mutagenesis reactions enable rapid insertion of PTM mimics to recombinant proteins with minor alteration on the modification site (e.g., single-atom variation (C to S) or loss of a stereocenter). These alterations were found to have a minor effect on protein integrity and activity [51]. This review covers recent developments in chemical protein mutagenesis and late-stage functionalization chemistry for the transfer of novel PTM mimicry into recombinant proteins while emphasizing novel transformations [52].

## 2. Late-Stage Cys Functionalization

Late-stage conjugation strategies allow facile protein diversification by rapidly transferring an array of unique modifications into proteins [53]. The unique nucleophilic nature and low abundance of Cys compared to other residues enables facile protein functionalization with various PTMs in high homogeneity [54]. In this strategy, the protein of interest is recombinantly expressed with Cys mutation at the desired modification site to allow chemoselective transfer of electrophilic PTM precursors. These approaches enable direct protein functionalization with “small” PTMs such as mono-/di-/trimethylation [47], succinylation [55], glycosylation [46], acetylation [56] marks, and large complex analogs (e.g., ubiquitylation and sumoylation) [57].

**Figure 2:**
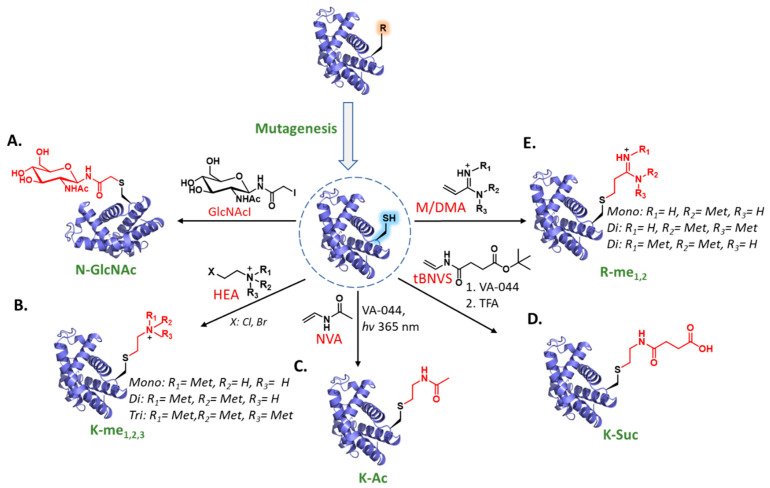
Chemical mutagenesis via late-stage Cys functionalization. Chemoselective incorporation of PTM mimics via direct Cys modification: (**A**) Asn glycosylation; (**B**) Lys methylation; (**C**) Lys acetylation; (**D**) Lys succinylation; (**E**) Arg methylation.

Early seminal work on protein functionalization with carbohydrates was conducted by Flitsch and coworkers using Cys alkylation chemistry (Figure 2A) [46]. Using a site-directed mutagenesis technology, the authors expressed target proteins with a single point mutation: Asn to Cys. Next, recombinant proteins were selectively glycosylated via the *S*-alkylation reaction using glycosyl-β-*N*-iodoacetamides (GlcNAcI). This approach enabled the synthesis of homogeneously glycosylated proteins that carried saccharide side-chains at programmed natural and unnatural positions. The authors employed this strategy to modify essential therapeutic targets such as erythropoietin (EPO) hormone with different *N*-glycosylation sites essential for its biological activity. Several EPO analogs with a single Asn-to-Cys mutation on native glycosylation sites (N24C, N38C, or N83C, individually) were successfully expressed and reacted with GlcNAcI in the presence of imidazole additive to achieve selective glycosylation only on Cys residue. Notably, these conditions enabled the production of the desired glycoprotein even in the presence of native protein disulfide bonds. It has also been shown via dynamic light scattering assay that the *N*-acetyl glucosamine residue did not considerably affect the stability of the muteins relative to the wildtype EPO.

A few years later, Shokat and coworkers applied the same concept to generate modified proteins with site- and degree-specific Lys methylation (Figure 2B) [47]. In this approach, proteins of interest (e.g., histone H3 analogs) were recombinantly expressed with Cys substituting the Lys residue at the modification site. Next, the authors installed several methylated Lys analogs (mono-, di-, and trimethylation) using different electrophilic 2-haloethylamine (HEA) analogs bearing the desired methylation state. To achieve selectivity, native Cys-110 in H3 protein was mutated to Ala without disrupting H3 folding and nucleosome function. Importantly, aminoethylcysteine has a minor structural alteration compared to Lys; substituting the lysine γ-methylene with a sulfide led to a slight lengthening of the side-chain. It has been demonstrated that methyl-Lys analog (MLA) side-chains do not significantly differ from the natural methyl-Lys epitopes, leading to the overall conclusion that MLAs are reasonable mimics to natural methyl-Lys residues as found in different binding assays. Several more functional tests were presented (e.g., binding and enzymatic assays), concluding that MLAs function similarly to natural Lys methylations in the context of histones PTM and nucleosome remodeling. This work proves the potential of MLA histones for investigating nucleosome-level properties of methylated nucleosomes. Although MLAs are stable, it is worth keeping in mind that they contain a thioether bond and are, therefore, sensitive to oxidation.

The success in transferring several PTMs via Cys alkylation with electrophilic alkyl halide substances has triggered the use of this approach to install acetyl-Lys into proteins. For instance, following the success of using aminoethylation of Cys residue to convert Cys to 4-thialysine as a functional mimic to methyl-Lys, a similar method was tested to prepare acetyl-Lys mimics [58]. However, this approach failed to provide the acetyl-Lys product with a feasible yield after several attempts of using *N*-acetyl-aminoethyl bromide/iodide or *N*-acetylaziridine for Cys alkylation [56,58]. Alternatively, the Cole group introduced the synthesis of acetyl-Lys mimic to generate methylthiocarbonyl-thiaLys (MTCTK) [58], a thiocarbamate analog of acetyl-Lys. This result was achieved by using Cys alkylation with methylthiocarbonyl-aziridine. Finally, the authors exploited this methodology to prepare site-specifically acetylated H3 analogs at three different positions (Lys-9, Lys-18, and Lys-27, individually) starting from recombinantly expressed H3 with Cys substituting a desired Lys residue at the modification site. It has been shown that even though MTCTK is not identical to the acetyl-Lys structure, it appears to maintain aspects of molecular recognition through protein binding and enzymatic regulation. In addition, it has been shown that this acetylation mimic has resistance to histone deacetylase cleavage, which may provide utility in the complex environments found in transcriptional and chromatin assays.

To facilitate the production of acetylated proteins with an ideal acetyl-Lys mimic, Liu and coworkers developed an effective insertion of acetyl-Lys mimic using a radical thiol–ene-mediated addition of Cys thiol (Figure 2C) [56]. The potential of this approach was investigated with several targets, including ubiquitin and histone H3 and H4 proteins with Lys to Cys mutation at positions 48, 16, and 36, respectively. Exposing each of these proteins to *N*-vinyl-acetamide (NVA) in the presence of the radical initiator VA-044 under UV irradiation at 365 nm furnished the desired acetylated product. Functional studies revealed that the generated acetyl analogs are a good functional mimic of the natural acetyl-Lys. In addition to Cys alkylation with an electrophilic alkyl halide, this work introduced the previously unexplored thiol–ene radical chemistry as an effective platform for the *S*-acetamidoethylation of Cys residues in recombinant proteins.

Jing et al. extended this thiol–ene chemistry for site-specific installation of succinyl-Lys (Ksuc) mimic into recombinant histone proteins (Figure 2D) [55]. This strategy was used to functionalize Lys 34 of H2B (mutated to Cys) using the *tert*-butyl ester of *N*-vinyl-succinamate (tBNVS) in the presence of VA-044 radical initiator. After this, *tert*-butyl deprotection using trifluoroacetic acid (TFA) furnished the desired succinated H2B. Structural and functional assays (such as antibody-specific recognition and hydrolysis of succinyl Lys via enzymatic reactions) confirmed that the Ksuc analog has similar properties to the native Ksuc, allowing investigation of the role of H2BK34suc in regulating nucleosome dynamics. This study, therefore, opens a new opportunity to examine the potential roles of histone Ksuc in regulating nucleosome and chromatin structure and dynamics.

Following the extensive work performed to install Lys PTM mimics, Fujimori and coworkers developed an effective platform to incorporate all three methylarginine analogs (MAAs) on recombinant protein with defined methylation status (Figure 2E) [59]. This method was based on Cys alkylation with α,β-unsaturated amidine scaffold with three degrees of *N*-methylation, as appears in the natural methylated Arg. This approach was exploited to prepare methylated histone H3 at Arg 2 and H4 at Arg 3, which were mutated to Cys to allow a conjugation reaction with the amidine precursors monomethylarginine (MMA) and dimethylarginine (DMA). The synthesized Arg analogs bear two main differences from the natural ones; γ-methylene is replaced with a sulfur, while ε-methylene replaces the secondary amine (as a result of using amidine-based reactant rather than guanidine). Several successful specific antibody Western blot (WB) analyses on different analog-modified histones indicated that MAAs are reasonable mimics of native methylarginine. The authors also examined the influence of asymmetric dimethylarginine analogs on nucleosome reconstitution and showed that they have a minimal effect on nucleosome reconstitution efficiency to form mono-nucleosomes.

**Figure 3:**
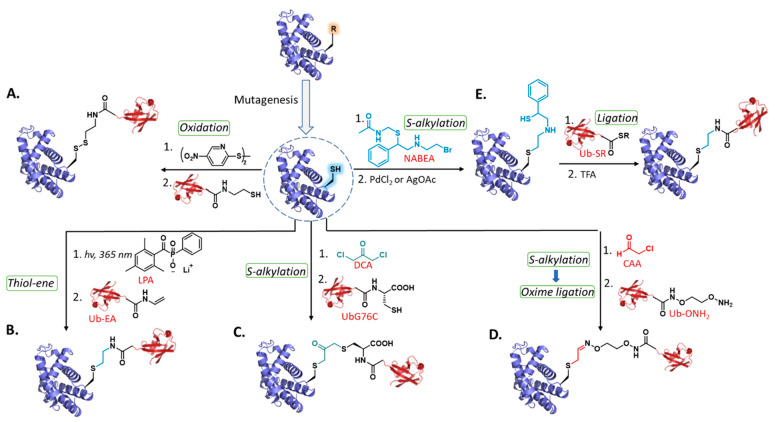
Chemical protein ubiquitylation. Chemoselective ubiquitylation: (**A**) oxidation; (**b**) thiol–ene addition; (**C**) *S*-alkylation; (**D**) *S*-alkylation coupled with oxime ligation; (**E**) *S*-alkylation coupled with NCL.

Protein ubiquitylation is an essential PTM that controls various vital cellular processes [60]. This modification involves an enzymatic cascade consisting of E1, E2, and E3, which collaborate to install the ubiquitin (Ub, 76 AAs) monomer to the Lys residue of a protein substrate. As described above, Cys alkylation has been extensively used to transfer small electrophilic PTM mimics into proteins. Recently, this method was expanded to transfer large and complex PTMs into recombinant proteins [61,62]. In this regard, several novel synthetic approaches were developed to install Ub unit into the protein of interest with high fidelity [57]. For example, early reports showed that Ub units could be connected to the target protein via a disulfide linkage (Figure 3A) [63,64]. However, the susceptibility of disulfide bonds in biochemical settings (e.g., reducing conditions) triggered the development of several other conjugation reactions with stable linkage, mainly by using bifunctional linkers and chemoselective ligation chemistry. Strieter and coworkers used thiol–ene chemistry to prepare several (poly)ubiquitylated variants (Figure 3B) [65]. The authors used recombinant protein expression technology to prepare proteins harboring a Cys residue instead of Lys via site-directed mutagenesis and Ub-bearing allylamine tethered to the C-terminus (Ub-EA). Reacting both proteins in the presence of lithium acyl phosphinate (LAP) photoinitiator and UV irradiation at 365 nm furnished the desired monoubiquitylated substrates through the thioether linkage. Encouraged by these results, several di- and triubiqutylated analogs were prepared in varied yields, starting from protein substrates with multiple Cys residues. Notably, it has been found that the accessibility of Cys residue within the substrate dramatically affects the efficiency of the conjugation reaction. Enzymatic studies via deubiquitinases (DUBs) enzymes revealed that the thioether–isopeptide bond is processed similarly to that of the native isopeptide linkage, thus indicating the potential of thiol–ene reaction in the generation of ubiquitylated proteins with amenable linkage. Biochemical analysis with the generated Ub analogs revealed that the orientation of the Ub unit has a critical effect on DUBs activity, indicating that (poly)ubiquitin topologies have a regulatory mechanism for linkage-selective interactions. This agrees with previous studies showing that Ub chains bearing different linkages or at different sites led to distinct structural and functional effects [61,62].

To generate stable linked Ub proteins, Long et al. introduced the synthesis of nonhydrolyzable Ub mimics using 1,3-dichloroacetone (DCA) [66,67] as a crosslinker and applied it to generate monoubiqutylated histone proteins (Figure 3C) [68]. To connect both proteins, a Gly-to-Cys mutation at position 76 was inserted into the Ub’s C-terminus (UbG76C), while the ubiquitylation site at Lys-119 in histone H2A was mutated to Cys. Using this design, the authors managed to crosslink both proteins via DCA and reconstitute histone dimers/octamers containing a stable Ub linkage. Furthermore, these analogs were successfully assembled to form monoubiqutylated nucleosomes, indicating that this method does not interfere with histones assembly and nucleosome reconstitution.

The combination of hybrid conjugation reactions has also been examined for sequential incorporation of multiple Ub units [69]. Brik and coworkers introduced a new strategy to generate a set of well-defined (poly)ubiquitylated proteins bearing an oxime and thioether linkage between the chain and the substrate (Figure 3D). The authors demonstrated the strategy on α-globin protein with a single Cys residue at position 104. The α-globin reacted with chloroacetaldehyde (CAA) to convert Cys-104 into an aldehyde, while the target Ub unit was modified with an oxyimino moiety at the C-terminus (Ub-ONH_2_). This design enabled the connecting of both proteins by *S*-alkylation chemistry followed by oxime ligation to provide monoUb-α-globin. Furthermore, combining oxime ligation with NCL using bifunctional Ub analogs enabled the preparation of di-, tri-, and tetraUb-α-globin. Notably, the authors found that the isopeptide replacement with an oxime–thioether linkage is resistant to DUBs and the proteasome. Extensive biochemical studies coupled with proteomics analysis with the (poly)ubiquitylated proteins achieved through this approach revealed insight into the different molecular signals of Ub chain length in proteasomal degradation [69].

Liu and coworkers reported a cysteine aminoethylation-assisted chemical ubiquitylation (CAACU) strategy for installing mono- and diUb into histone analogs and a small ubiquitin-like modifier (Figure 3E) [70]. The authors first reacted *N*-alkylated protected 2-bromoethylamine (NABEA) with recombinant histone proteins with Lys to Cys mutations (e.g., histone H2BK34C). Following the alkylation step, the thiol protecting group was removed to enable auxiliary mediated NCL with the Ub thioester (Ub-SR) to yield the ubiquitylated histone conjugate. Finally, removing the auxiliary via TFA provided the desired modified analogs with thioether linkage. The modified histones with thioether–isopeptide bonds were chemically stable and could be readily reconstituted into the native nucleosomal context for structural and activity studies.

## 3. Dehydroalanine Coupled via Michael Addition Chemistry

Protein labeling has traditionally relied on nucleophilic amino-acid residues such as Cys and Lys for chemoselective manipulations with electrophilic reagents. These approaches allowed rapid protein modification with a broad set of natural and non-natural residues, including PTMs mimics. However, site selectivity remains the main limitation of these methods. Therefore, there is growing attention toward incorporating a novel reactive functionality at a predetermined site for general and selective protein modifications. The ability to introduce the nonproteinogenic amino acid Dha into proteins has attracted several groups because of its robust and selective reactivity toward thiol and amine nucleophiles, which provides a novel electrophilic site amenable to varied transformations [71]. The insertion of Dha functionality into recombinant proteins can be achieved in a stepwise manner by incorporating orthogonal residue using site-directed mutagenesis technology or through genetic incorporation method, followed by chemoselective elimination to convert the desired residue to Dha. Different protocols have been reported for effective Dha insertion into proteins, usually under mild and biocompatible conditions [51]. These methods are based on β-elimination of Cys, Sec, or phosphorylated serine (pSer) residues to yield Dha as an orthogonal handle for conjugation addition. Introducing Dha into proteins enabled protein modifications with a broad range of thiolated PTM mimics through Michael-type addition chemistry. This strategy enables effective insertion to access an array of PTM mimics, including mono-, di-, trimethylation, phosphorylation, glycosylation, acetylation, and ubiquitylation.

**Figure 4:**
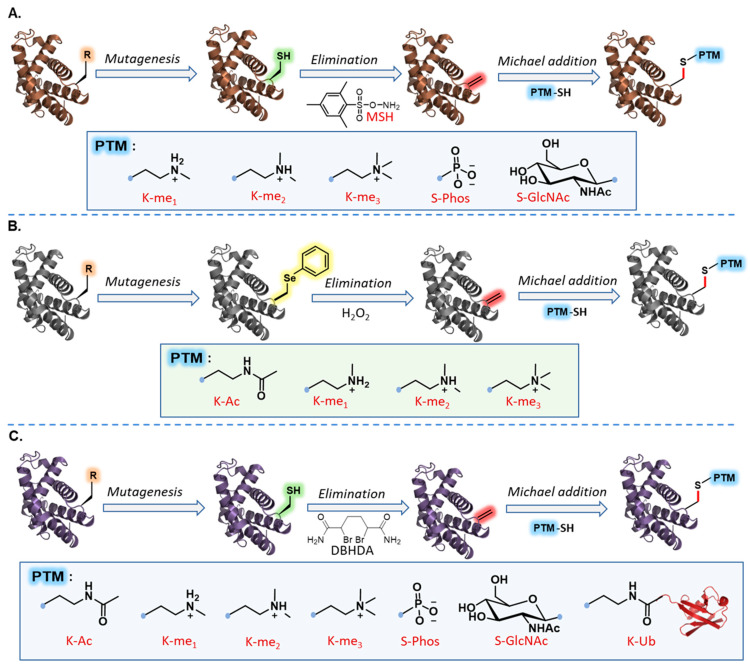
Chemical mutagenesis via dehydroalanine coupled via Michael addition chemistry. (**A**) Chemoselective transfer of multiple PTM mimics via Cys elimination using MSH followed by Michael addition: mono-/di-/trimethylation, phosphorylation, and glycosylation. (**B**) Chemoselective transfer of PTM mimics to Lys via Sec elimination followed by Michael addition: mono-/di-/trimethylation and acetylation. (**C**) Chemoselective transfer of multiple PTM mimics via Cys elimination followed by Michael addition: mono-/di-/trimethylation, phosphorylation, glycosylation, acetylation, and ubiquitylation.

In 2008, Davis and coworkers reported a facile and rapid protocol for Dha formation on recombinant proteins via oxidative elimination of Cys residue using *O*-mesitylenesulfonylhydroxylamine (MSH) reagent (Figure 4A) [44]. The potential of this approach was investigated on Subtilisin protein with a single Cys residue. Using MSH, the authors converted Cys to Dha within minutes under an aqueous buffer in alkaline conditions. The installed Dha handle enabled the practical addition of several thiol nucleophiles bearing PTM analogs into the target protein, including Lys methylation, Ser phosphorylation, and Ser glycosylation (GlcNAc). Importantly, modified Subtilisin protein was found to be catalytically active after both synthetic steps, indicating preserved protein integrity under the elimination and conjugation reactions. This report introduced the potential of Dha chemistry to transfer multiple PTM mimics into recombinant proteins with minimal alteration on the modification site.

Using the same concept, Schultz and coworkers introduced an alternative strategy to introduce Dha into recombinant proteins through the genetic incorporation of the unnatural amino acid phenylselenocysteine (PhSec), followed by elimination using hydrogen peroxide (Figure 4B) [72]. Michael-type addition reactions with the corresponding thiols were performed to transfer methyl and acetyl-Lys analogs into histone H3 protein, which was found to function similarly to their native counterparts. While both described approaches provide a facile method to obtain homogeneously modified proteins with a wide range of PTM mimics, the use of MSH and hydrogen peroxide reagents to convert Cys or Sec to Dha, respectively, has been found to oxidize native amino-acid residues (e.g., Met, His, Asp, Glu, and Lys). These limitations triggered the development of milder and more effective reaction conditions to insert Dha with minimal alteration of protein sequence and integrity.

Several strategies have been developed to incorporate Dha into recombinant proteins under biocompatible conditions [71]. Davis and coworkers reported a facile conversion of Cys to Dha using a bis-alkylation reaction followed by thiol elimination under slightly alkaline conditions. The authors introduced the commercially available and water-soluble reagent 2,5-dibromohexanediamide (DBHDA) as an excellent reagent for Dha formation starting from engineered Cys residue (Figure 4C) [71]. The facile introduction of Dha into recombinant proteins and the unique electrophilicity of α,β-unsaturated carbonyl moiety enabled the transfer of numerous PTM mimics via Michael-type addition reactions with various nucleophiles. Chalker et al. demonstrated the potential of the Dha site to prepare synthetic histone proteins bearing diverse PTM mimics [73]. The authors mutated the desired modification site in histone H3 to Cys using site-directed mutagenesis and then converted it to Dha using DBHDA. The introduction of the electrophilic moiety enabled rapid and effective insertion of all three methylation states of Lys, acetylated Lys, phosphorylated, and glycosylated Ser mimics by adding the appropriate thiol (Figure 4C). Furthermore, the sequential formation of Dha and its conjugation with target PTM mimics have been successfully achieved in a simple one-pot operation to provide the final modified products in good yield.

The robustness of Dha chemistry has also enabled the transfer of complex PTM mimics into large macromolecules for biochemical studies. For example, to elucidate the molecular basis of the histone *O*-GlcNAcylation in epigenetic regulation and gene transcription, Lercher et al. generated site-specifically GlcNAcylated histone H2A at Thr-101 using DBHDA-mediated Dha formation followed by Michael addition reaction with a thio-GlcNAc reagent [74]. Nucleosome assembly with GlcNAcylated histone H2A followed by structural and functional analysis revealed that H2A GlcNAcylation can modulate chromatin structure by directly destabilizing H2A/H2B dimers in the nucleosome. The same group used the Dha approach to incorporate GlcNAcylated histone H2B at Ser-112 to generate site-specifically modified nucleosomes [75]. Proteomic analyses revealed a direct interaction between GlcNAcylated H2B and the facilitates chromatin transcription (FACT) complex and suggested to trigger FACT-driven transcriptional control.

Dha chemistry has been used extensively to transfer PTM mimics into proteins using small-molecule thiols, as described above (Figure 4) [76,77]. To expand the applicability of Dha chemistry for installing large and complex modifications, Brik and coworkers demonstrated a strategy for chemoselective protein ubiquitylation using Dha to prepare ubiquitin conjugates bearing a close mimic of the native isopeptide bond (Figure 4C) [78]. The authors used α-globin protein as a model system by first converting Cys-104 to Dha using DBHDA, followed by Michael addition with the thiolated C-terminal peptide fragment of Ub. NCL reaction with the conjugated peptide enabled the transfer of full-length Ub into α-globin protein. This work demonstrated the expansion of Dha chemistry to transfer large PTMs into proteins in an effective manner. In addition to its broad utility in chemical protein modifications, the facile incorporation of Dha has demonstrated broad utility in total chemical protein synthesis and protein semi-synthesis to prepare novel activity-based probes of ubiquitylated proteins for structural and functional studies.

## 4. Carbon–Carbon Bond-Forming Reactions

**Figure 5:**
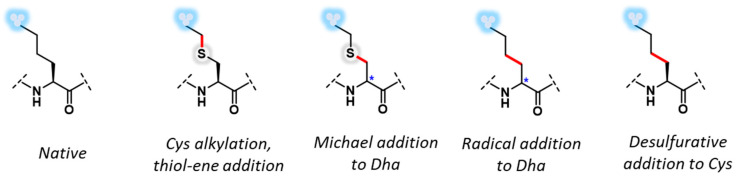
Synthetic strategies for site-selective installation of PTMs.

The vast majority of current chemical transformations to install PTMs rely on unnatural carbon–heteroatom linkages (e.g., Cys and Dha conjugation addition chemistry) (Figure 5). While such methods enable PTMs to be investigated, methods that form the native modification are ideal. Access to synthetic transformations via carbon–carbon bond-forming reactions enables PTM installation with “perfect” connectivity by forming a native side-chain linkage. However, the ability to transfer PTMs to protein with minimal alteration represents a challenge due to the need for chemical handles with unique reactivity to allow chemoselective covalent bond formation.

**Figure 6:**
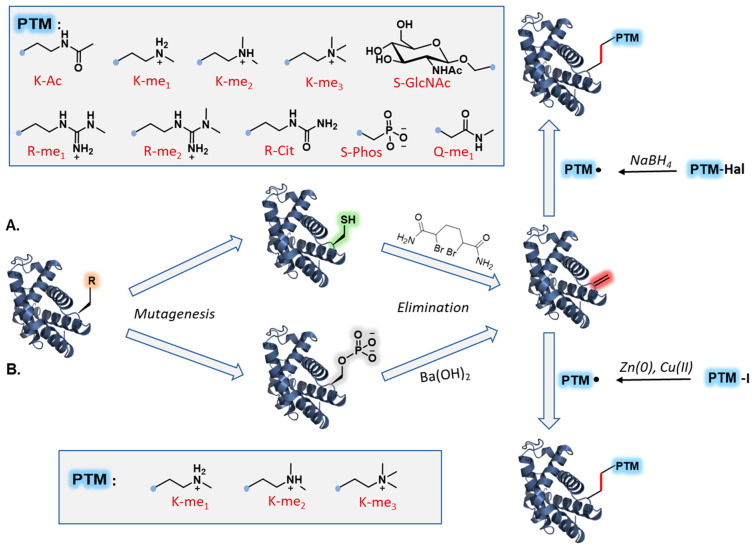
Chemical mutagenesis via carbon–carbon bonding reactions. (**A**) Chemoselective transfer of multiple PTM mimics via Cys elimination followed by radical additions: mono-/di-/trimethylation, acetylation, citrullination, glycosylation, and phosphorylation. (**B**) Chemoselective transfer of Lys PTM mimics via pSer elimination followed by radical additions: mono-/di-/trimethylation.

In 2016, the Davis and Park groups separately reported a brilliant strategy for modifying recombinant proteins on a preinstalled Dha site using radical-mediated β,γ carbon–carbon forming chemistry (Figure 6) [79,80]. This versatile technique enabled selective protein functionalization at a programmed Dha tag with a set of transformations with minimal alteration on the modification site. Davis and coworkers developed C(*sp^3^*)–C(*sp^3^*) bond-forming reactions through carbon free-radical chemistry under biocompatible conditions (Figure 6A) [79]. Unlike traditional two-electron chemistry, this method is based on water-tolerated free radicals in the presence of NaBH_4_/In^(0)^ and alkyl halides (R-Hal) through the generation of single-electron species via C–Hal homolytic bond division or through single-electron transfer. The designed reaction enabled a wide diversity installation of native, non-native, and PTMs mimics into recombinant proteins, such as phosphorylated, hydroxylated, methylated, and glycosylated analogs, with excellent site and regioselectivity. To prevent side reactions, using NaBH_4_ as a quenching reagent and removing molecular oxygen from the buffer solutions have proven to be essential. The versatility of this strategy was demonstrated in the construction of >25 modified side-chains on different protein targets. Importantly, this approach revealed insight into the biological functions of essential PTMs, such as glycosylation, phosphorylation, and methylation. For example, synthetic nucleosomes with site-specifically methylated Lys and Arg residues were prepared via this method, including radiolabeled analogs. Spectroscopic and cellular studies revealed insight into the functional mechanistic role of the methylation mark with other associated proteins (e.g., readers and erasers).

Using the same concept, Park and coworkers designed a “three-step” strategy to transfer Lys-methylation mimics into recombinant proteins (Figure 6B) [80]. The authors first introduced pSer through an orthogonal *E. coli* translation system to furnish pSer-containing recombinant proteins. Then, the PSer residue was converted by phosphate elimination to Dha using Ba(OH)_2_. Finally, conjugate addition of alkyl iodides to Dha, promoted by Zn(II) and Cu(II), enabled chemoselective C(*sp^3^*)–C(*sp^3^*) bond formation on target proteins. The applicability of this approach was exploited to modify several proteins, including different methylation states of histone H3 at Lys79. The homogeneous histone H3 analogs elucidated the effect of H3K79 methylation on chromatin transcription, which was found to stimulate transcription through histone acetylation via the transcriptional coactivator p300. Notably, H3K79 methylation states differently affect p300-mediated chromatin acetylation, indicating a potential regulatory role of the methylation level in the gene transcription process. Similar to the report by Davis and coworkers, the conjugation addition reaction is believed to be mediated through radical chemistry. Both reports offer a powerful platform to install PTMs with unique connectivity and close mimicry to native counterparts to decipher their molecular role.

**Figure 7:**
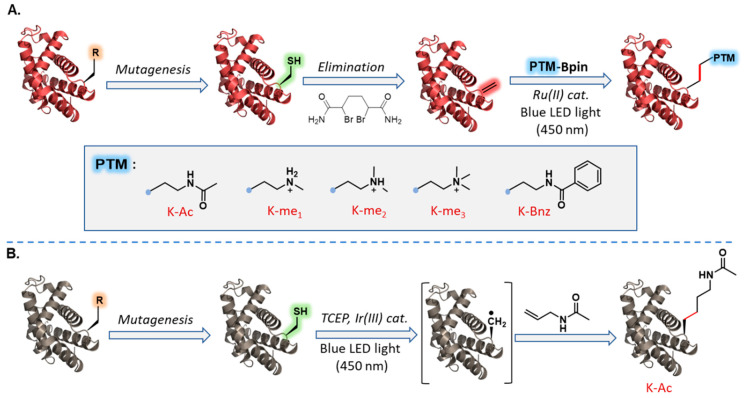
Chemical mutagenesis via visible-light-mediated carbon–carbon bond formation. (**A**) Chemoselective transfer of multiple Lys PTM mimics via Cys elimination followed by visible-light-driven Dha functionalization: mono-/di-/trimethylation, acetylation, and benzylation. (**B**) Chemoselective transfer of native acetyl-Lys via visible-light-mediated desulfurative carbon–carbon bond formation approach.

In 2020, Davis and coworkers introduced a robust alternative strategy to incorporate a range of challenging functional groups and PTMs into recombinant proteins through carbon–carbon bond formation using a single-electron transfer method (Figure 7A) [81]. The authors described a visible-light-driven installation of several natural and non-natural side-chains, as well as PTMs, at Dha-tagged proteins under mild conditions. The Dha-tagged proteins reacted in the presence of Ru(II) photocatalysts with blue LED light (450 nm) to enable carbon–carbon bond formation in high fidelity. Like the previous methods, the reaction was driven by radical generation from radical precursors such as boronic acid catechol ester or pyridylsulfonyl derivatives coupled with Fe(II). These conditions enabled the versatile transfer of a broad range of native and modified amino-acid side-chains (>50 unique analogs) into a set of recombinant proteins. Importantly, this approach allowed for the insertion of several PTM analogs into histone proteins with acetyl-, methyl-, and benzyl-Lys mimics to probe the interaction of posttranslational enzymes (e.g., readers and erasers) and modified histones.

The development of carbon–carbon bond-forming reactions enabled protein functionalization with closer mimicry to the natural PTMs compared to the C–S bond formation as presented in the *S*-alkylation of Cys and Michael addition to Dha (Figure 5). Althoughsuch methods solved the issue of the noncanonical C–S bond, current C–C bond-forming reactions provide desired products with loss of native stereochemistry at the modified residue (Figure 5). While these seminal studies enable the investigation of the biological outcome of several PTMs in complex systems (e.g., histones), synthetic methods that lead to the native modification have also been investigated. Mitchel and coworkers reported a site-selective installation of ε-amine modifications into peptide and protein via a visible-light-mediated desulfurative carbon–carbon bond formation method (Figure 7B) [82]. This reaction is mediated via the desulfurization of Cys to generate an alanyl-radical intermediate trapped in situ with an appropriately modified allylamine with retention of stereochemistry at the modified residue. This approach was extensively investigated to selectively install Lys-PTMs through native C(*sp^3^*)–C(*sp^3^*) bond formation. The reactions were performed using functionalized *N*-allyl analogs to directly modify Cys residue in the presence of Ir(III) photocatalyst when irradiated under blue light. The conjugation reactions were conducted using tris(2-carboxyethyl)-phosphine (TCEP) additive to facilitate the desulfurization process to generate reactive alanyl radical through phosphoranyl radical intermediate (Figure 7B). While several native Lys PTMs analogs were inserted into synthetic peptides using this method, only the acetylation mark was installed into recombinantly expressed protein (Ub at Lys 48). This work represents the first example of site-selective installation of Lys PTMs into proteins through a native carbon–carbon bond without disrupting the stereochemistry of the target residue. The potential of this approach to transfer other important PTMs remains to be investigated.

## 5. Conclusions and Outlook

Synthetic tools have been increasingly employed to modify protein using natural and non-natural modifications. This practice has dramatically expanded the capability to generate complex and uniquely modified proteins for functional analysis. This review has highlighted the versatility of current synthetic strategies to functionalize proteins with essential PTMs. Most reactions are capitalized on Cys residue as a modification site either through direct functionalization or in a stepwise manner via converting it to Dha followed by Michal addition [42,51]. These approaches enable precise and rapid installation of several PTM mimics into recombinant proteins. The molecular code of, for example, glycosylation, methylation, phosphorylation, acylation, and ubiquitination can, therefore, be studied in proteins of interest. Notably, current strategies rely on the formation of unnatural carbon–heteroatom linkage due to the need for orthogonal chemical handles for chemoselective covalent bond formation. The non-natural connectivity often achieved in Cys and Dha conjugation chemistry leads to slight lengthening of the side chain or loss of a stereocenter (formation of epimeric (l-/d-) mixtures in the case of Dha chemistry). While these alterations have been found to have a minor effect on protein integrity and activity, opportunities remain for the development of new synthetic strategies to transfer native PTMs through natural linkage and preserved chirality [51]. For example, recently, Dha chemistry has dramatically evolved to enable the installation of novel PTMs into proteins through natural linkage using carbon–carbon bond-forming chemistry. However, controlling the stereochemical outcome using this chemistry is still challenging [80,81]. More recently, a visible-light-mediated desulfurative carbon–carbon bond formation at Cys residues was introduced, which enables the incorporation of native PTMs with retention of stereochemistry at the modified residue [82]. This approach was extensively investigated to transfer Lys PTMs mainly to peptides; thus, the applicability of this approach to transfer PTMs to other residues remains to be explored. While novel transformations have been achieved using state-of-the-art methods to decipher fundamental biological systems, the regiospecificity of current chemical tools is still limited. The main challenge of these methods is the complexity of modifying a specific Cys residue in the presence of other reactive sites. Some reports demonstrated the ability to modify a specific solvent-exposed site/sequence but still lack generality [83,84]. Recent developments in the selective incorporation of Dha residue into Cys-rich protein [85] can, in principle, provide an opportunity to install PTMs in a site-specific manner. However, transferring different PTMs into the same protein is still challenging. Therefore, selective, and controllable strategies are required to expand the available toolbox for the production of novel complex proteins. Chemical protein synthesis provides a powerful platform to prepare homogeneously modified proteins by allowing selective insertion of virtually any desired PTM in a highly controlled manner. Recent developments in the field of protein synthesis, such as flow-based protein synthesis, chemoselective ligation technology, and post-synthetic modifications, have enabled the production of large and complex protein targets [86,87,88]. Thus, combining these synthetic strategies with molecular biology and enzymatic approaches is anticipated to extend the scope of protein production with desired PTMs to decipher their molecular code.
